# Resting Heart Rate Variability Measured by Consumer Wearables and Its Associations with Diverse Health Domains in Five Longitudinal Studies

**DOI:** 10.3390/s25237147

**Published:** 2025-11-22

**Authors:** Raymond Hernandez, Stefan Schneider, Herman J. de Vries, Jason Fanning, Dominic Ehrmann, Haomiao Jin, Raeanne C. Moore, Shannon Juengst, Aaron Striegel, Jack P. Ginsberg, Norbert Hermanns, Arthur A. Stone

**Affiliations:** 1 Center for Economic & Social Research, Center for Self-Report Science, University of Southern California, 635 Downey Way, Los Angeles, CA 90089, USA; schneids@usc.edu (S.S.); arthuras@usc.edu (A.A.S.); 2 Department of Psychology, University of Southern California, Los Angeles, CA 90089, USA; jginsberg@saybrook.edu; 3 Leonard Davis School of Gerontology, University of Southern California, Los Angeles, CA 90089, USA; 4 Department of Learning and Workforce Development, Netherlands Organisation for Applied Scientific Research (TNO), 2509 JE Soesterberg, The Netherlands; herman.devries@tno.nl; 5 Department of Health and Exercise Science, Wake Forest University, Winston-Salem, NC 27109, USA; fanninjt@wfu.edu; 6 Diabetes Research Institute of the Diabetes Academy Mergentheim, 97980 Bad Mergentheim, Germany; ehrmann@fidam.de (D.E.); hermanns@fidam.de (N.H.); 7 Department for Clinical Psychology and Psychotherapy, University of Bamberg, 96045 Bamberg, Germany; 8 School of Health Sciences, University of Surrey, Guildford GU2 7YH, UK; h.jin@surrey.ac.uk; 9 Department of Psychiatry, University of California San Diego, San Diego, CA 92093, USA; r6moore@health.ucsd.edu; 10 Brain Injury Research Center, TIRR Memorial Hermann, Houston, TX 77030, USA; shannon.juengst@utsouthwestern.edu; 11 Department of Physical Medicine & Rehabilitation, UT Southwestern Medical Center, Dallas, TX 75390, USA; 12 Department of Computer Science & Engineering, University of Notre Dame, Notre Dame, IN 46556, USA; striegel@nd.edu; 13 Departments of Research and Applied Psychophysiology, Adjunct Faculty, Saybrook University, Pasadena, CA 91103, USA

**Keywords:** heart rate variability, resting HRV, wearables, smartwatch, smartring, heart rate chest strap, digital biomarker

## Abstract

**Highlights:**

**What are the main findings?**

- Resting HRV as measured by consumer wearables either upon waking or while sleeping had small-to-moderate associations with more clinically oriented and trait-like (or slow-changing) health measures like average blood glucose, depressive symptoms, and sleep difficulty.
- Within one person, in one study we found that higher resting HRV was significantly associated with more recovery time from work, less mental exhaustion, and less alcohol consumption on the day prior; however, across studies, within-person correlations with prior-day general stress and mood measures were non-significant.

**What is the implication of the main findings?**

- A myriad of HRV metrics can be computed from wearables, but resting HRV measured upon waking or while sleeping may deserve greater attention as a potential measure of general health

**Abstract:**

Heart rate variability (HRV) is widely recognized as an indicator of general health, particularly time domain measures like the root mean square of successive differences (RMSSD) between consecutive heartbeats. Consumer wearables measuring HRV have potential for wide accessibility meaning that their broad use to capture HRV as a health biomarker is possible. Our objective was to investigate the validity of HRV measured by wearables as a general health indicator. We examined whether resting HRV assessed by wearables across five studies—two using smartwatches, two using heart rate chest straps, and one using a smartring—exhibited expected associations with diverse health domains, including mental, physical, behavioral, functional, and physiological. We focused on resting HRV measures recorded while in primarily stationary conditions, either upon waking or while sleeping, because such measures would theoretically reduce the effects of potential confounders such as movement artifacts, daytime caffeine intake, and postural changes. Wearables measured resting HRV had small-to-moderate associations with more clinically oriented and trait-like (or slow-changing) health measures like Hba1c (average blood glucose, *r* = −0.21, *p* = 0.014), depressive symptoms (*r* = −0.22, *p* = 0.024), and sleep difficulty (*r* = −0.11, *p* = 0.003). Wearable-measured resting HRV can potentially serve as a health biomarker, but further research is needed.

## 1. Introduction

Heart rate variability (HRV), the variation in time between consecutive heartbeats, reflects the adaptability of regulatory systems to stressors and challenges [1]. These systems include the autonomic nervous system (ANS) and the highly interrelated respiratory system, both of which influence HRV [1]. The sympathetic nervous system increases vigilance and heart rate in stressful situations, while the parasympathetic nervous system promotes restful and relaxed states, including decreased heart rate. Prolonged sympathetic activation has been associated with lower resting HRV, while more frequent parasympathetic activity has been associated with greater resting HRV. The respiratory system also influences HRV through respiratory sinus arrythmia (RSA), a reflex where heart rate increases when inhaling and decreases when exhaling. HRV biofeedback interventions leverage this reflex by training individuals to breathe at a rate that maximizes fluctuations in heart rate (e.g., six breaths per minute), with a variety of potential benefits for emotional regulation and cardiovascular health [2].

HRV, particularly time domain measures like the root mean square of successive differences (RMSSD), is increasingly recognized as an indicator of general health that is sensitive to strain experienced from stressors and other demands [1]. A low resting HRV, frequently viewed as a sign of a hypoactive parasympathetic system, has been linked to worse prognosis in cancer progression [3] and to several cardiovascular risk factors, including hypertension, smoking, and physical inactivity [4]. Meta analyses indicate that people with anxiety or depression have lower HRV compared to healthy controls [5,6]. Greater work stress and job strain have been associated with reduced HRV [4]. The exact mechanisms linking HRV to general health require further study, but people with poorer health—due to behavioral, genetic, and/or environmental factors—may exhibit impaired autonomic and cardiovascular modulation, reflected in lower HRV [4].

While current wearables appear to have accuracy in terms of measuring HRV that is comparable to ECG (under stationary conditions) [7,8,9], are much more user friendly than ECG, and have been used to inform the training of athletes, more research is needed to comprehensively examine what health domains, if any, HRV metrics captured via mobile technologies are associated with. Thus far, several relevant studies have been conducted. They are quite diverse with regard to characteristics such as the population of focus, the types of HRV metrics utilized, the statistical models used, and the health domains of interest. A prior study on U.S. knowledge workers, who have jobs involving developing and using knowledge rather than producing goods or services (e.g., consultants, administrators, and engineers), found that HRV computed between 8 am and 6 pm from a wrist wearable had a small but significant association with a momentary report of stress in the same day, and the authors concluded that wearable-derived HRV alone might not be sufficient to detect stress in naturalistic settings [10]. In adults with type 1 diabetes, greater average daily median HRV from heartbeat data collected by smartwatches over the whole day was found to be associated with lower diabetes distress, hyperglycaemia, and Hba1c, but not associated with depression, glycaemic variability, or hypoglycemia [11]. Among student interns, HRV measured upon waking for a two minute period with a chest strap was found to be predictive and indicative of stress and mental exhaustion [12]. In a study focused on a sample of U.S. adults, a majority of which had a past traumatic brain injury, HRV was assessed with a chest strap for a five minute period upon waking [13]. For approximately one quarter of this sample, greater HRV was found to be associated with lower negative affect, executive dysfunction, and fatigue. Finally, in a large sample of U.S. college students, greater nighttime HRV as measured by a smartring was associated with a lower chance of having experienced moderate-to-high stress [14].

### Present Study

These five aforementioned studies all have contributed to current understandings of relationships between wearable-measured HRV and health in different domains, but *we leverage their data further to examine the potential for wearable-measured HRV (measured upon waking or while sleeping) to act as a digital health biomarker*. If wearable-measured HRV is a digital biomarker for health, then we would expect measures of different domains of health to impact subsequent wearable-measured HRV within a person (e.g., excessive stress or blood sugar on the day prior should lower it), and that measures of different health domains would be associated with average HRV across the study (e.g., greater average stress or blood sugar should decrease it). Based on prior evidence, we specifically expected lower HRV to be associated with the poorer scores in the following health domains both within and between individuals: mental health, physical health, behavioral health, physiological health, and functional health [4,5,6,15]. Note that the conceptualization of health used here was intentionally broad to allow examination of a wide range of outcomes and reflect the multi-faceted nature of health. Also note that though exposure to an overwhelming amount of stress can also result in greater HRV, a phenomena known as “parasympathetic rebound” [16], such effects are often seen in specialized populations like long-distance truck drivers [17] and on-call physicians [16]. Such populations were not the focus of the aforementioned studies.

An advantage of secondary data analysis is that it allows us to examine health correlates of wearable-measured HRV without the time and expense of carrying out a new study [18]. Longitudinal observational studies, especially those involving repeated completion of surveys, are often expensive [19] due to costs for the required devices, staff to train participants and monitor their compliance over the study period, and staff to manage the incoming data. This may be why wearables-focused studies with a component of repeated self-reporting often have much smaller sample sizes than studies where only baseline characteristics and information passively recorded by wearables are collected, which can have millions of individuals represented [20]. A disadvantage of secondary data analysis is that researchers have no control over which individuals were sampled, which variables were collected, and overall design [18]. However, researchers may use insights gained from secondary analysis to generate more refined hypotheses [21] that can inform decisions about the design of a future study.

## 2. Methods

### 2.1. Resting HRV from Wearables as a Digital Biomarker

A variety of devices can assess HRV. The gold-standard approach for measuring HRV is the use of multi-lead electrocardiograms (ECGs) [22], but this necessitates specific placement of electrodes (typically 12) and the use of expensive equipment often only available in medical settings that makes consumer use unfeasible. Heart rate chest straps are single-lead with only two built in electrodes, offer a more affordable and home accessible alternative to multi-lead ECG if the primary interest is HRV measurement, and have been validated against the gold standard ECG [23], but they can be uncomfortable to wear over longer periods. Wrist and finger wearables are more convenient and cost-effective, but evidence collected from a variety of device models suggests that their HRV measurements approximate ECG primarily in stationary, but not moving, conditions [7,8,9,24]. This is because HRV from wrist and finger wearables is recorded via photoplethysmography (PPG), the light-based detection of peripheral blood flow to capture cardiovascular activity [25], and PPG is very susceptible to motion artifacts such that several prior studies have found poor associations between PPG-based pulse rate variability and HRV [24,26]. In any case, even if HRV could be accurately assessed in non-stationary conditions, the impact of physical activity can cloud any links to cognitive, emotional, social and health processes [22]. Thus, researchers are often more interested in HRV measured in stationary, not moving, conditions [22].

“Resting” HRV is a potentially useful measure that can be captured by wearables with some applied use as a digital biomarker [27,28]. Resting HRV measures are those taken while people remain stationary (e.g., seated or lying supine). One way it is commonly assessed is with HRV recordings taken for one-to-five minute periods after waking, using technologies such as a chest strap or smartphone camera [22,29]. Another common method is recording HRV at nighttime while a person is asleep [30]. An attractive feature of waking and nighttime resting HRV measures specifically is that they are less likely to be confounded by the myriad of other factors that can affect HRV including daytime alcohol intake, caffeine, smoking, and postural changes [22,31,32]. In athletes, resting morning HRV measures assessed daily have often been used to inform their training, where HRV values significantly lower than a person’s normal baseline HRV can indicate excessive training load on the prior day [27,28]. From here on, our use of the term “resting HRV” refers to resting HRV measured upon waking or while asleep.

### 2.2. Datasets

Our focus was on health correlates of resting HRV as measured by consumer wearables, so included studies needed to have wearables measuring resting HRV or the raw data to compute it, as well as at least one measure pertinent to one of the various domains of health. Additionally, datasets needed to be available for secondary analyses (i.e., publicly available or accessible upon request from the investigators). Five broad health domains were considered: (1) mental health and emotions, (2) physical symptoms and stress, (3) health behaviors, (4) everyday functioning, and (5) physiological markers. The study was approved by the University of Southern California’s Institutional Review Board. All study procedures were performed in accordance with the relevant guidelines and regulations of the IRB, and in accordance with the ethical standards laid down in the 1964 Declaration of Helsinki and its later amendments.

#### 2.2.1. Study 1: U.S. Knowledge Workers

The largest dataset analyzed came from “Project Tesserae”, which included 717 U.S. knowledge workers who completed an ecological momentary assessment (EMA) survey once daily for approximately two months, with survey times rotating between 8 am, noon, and 4 pm [33]. Only participants with at least one night of HRV data were included in the analyses (717/757, 95% of sample). Participants wore a Garmin Vivosmart 3 smartwatch (Garmin, Olathe, KS, USA), from which interbeat interval (IBI) data (i.e., time between heartbeats) was extracted using a modified version of the Student Life app [34]. They also completed a battery of baseline surveys. Participants on average had 25.6 (SD = 19.9) nights of HRV data recorded. EMA questions asked about stress, anxiety, positive affect, and negative affect with the general structure “How would you rate your current level of X?” Baseline surveys considered were the Positive and Negative Affect Schedule (i.e., positive and negative affect over the past few weeks) [35], trait aspect of the State-Trait Anxiety Inventory [36], Pittsburgh Sleep Quality Index (higher score indicating worse sleep quality, with 5 or higher corresponding with significant sleep difficulties) [37], the International Physical Activity Questionnaire [38], the neuroticism personality subscale of the Big Five Inventory [39], and Shipley cognitive health tests (which assess crystallized and fluid intelligence) [40].

#### 2.2.2. Study 2: German Adults with Type 1 Diabetes

The second dataset came from the “Towards a Better Understanding of Diabetes Distress, Depression and Poor Glycaemic Control Leading to Personalised Interventions for People With Diabetes (DIA-LINK)” study [41]. Data from 108 of 203 DIA-LINK participants who provided at least one night of HRV data were included in the analyses. They had an average of 6.0 (SD 4.2) nights of recorded HRV data. All participants had type 1 diabetes and were recruited from a diabetes clinic in Germany with quota sampling to ensure that at least 75% of the sample had elevated diabetes distress and/or depression symptoms [41]. Participants were asked to complete 4 EMA surveys daily for 17 days, wore a Garmin Vivosmart 3 smartwatch, had IBI data extracted from the smartwatch with the Ilumivu app, and underwent baseline assessments of physiological biomarkers. Health-relevant EMA questions analyzed asked about stress, mood, and energy four times a day. Work stress, family stress, diabetes distress, people stress, and diabetes energy were asked about at the end of the day. Baseline questionnaires considered in this study were the Patient Health Questionnaire (depression) [42], the Center for Epidemiologic Studies Depression Scale (depression) [43], the Resilience Scale [44], the Problem Areas in Diabetes Scale (diabetes distress) [45], and the Diabetes Self-Management Questionnaire [46]. Other health relevant baseline measures utilized in analyses were self-reported neuropathy, retinopathy, and smoking status. Physiological measures examined were cholesterol, triglycerides, HDL (high-density lipoprotein cholesterol), LDL (low-density lipoprotein cholesterol), Hba1c (Hemoglobin A1c), IL6 (Interleukin-6), IL10 (Interleukin-10), and TNF (tumor necrosis factor).

#### 2.2.3. Study 3: Student Interns from The Netherlands

The third dataset analyzed contained information from 25 students (primarily young adult females) in the Netherlands who were about to start their first full-time internship in applied psychology, social work, or physiotherapy [12]. For 15 weeks they were asked to complete daily surveys and to record their resting HRV upon waking with a Polar H7 chest strap (Polar Electro, Kempele, Finland) and the Elite HRV application (Elite HRV, Asheville, NC, USA) for a two-minute recording period. On average, they had 60.0 mornings (SD 33.7) of HRV data recorded. End-of-day surveys covered the whole day and asked about the level of demands experienced, stress, recovery time from work, detachment from work, level of energy, perceived meaning/purpose of activities, and mental exhaustion. Morning surveys asked about sleep quality and alcohol consumption on the day prior. Twice-daily EMA surveys (once in the morning and again in the evening) addressed momentary feelings of fatigue, fitness, happiness, vigor (i.e., feeling like undertaking things), and self-efficacy.

#### 2.2.4. Study 4: U.S. Adults with Lifetime History of Traumatic Brain Injury

The fourth dataset analyzed included 55 U.S. adults (44 with a history of traumatic brain injury, TBI) who completed baseline surveys, two weeks of daily surveys, and daily 5 min resting HRV measures upon waking with a Polar H10 chest strap (Polar Electro, Kempele, Finland) and the Elite HRV mobile application [13]. On average they had 12.8 mornings (SD 1.8) of HRV data recorded. The items in the daily surveys were from the Behavioral Assessment Screening Tool for Mobile Health (BAST mhealth) [47] and asked about negative affect, fatigue, executive functioning, impulsivity, and substance misuse over the past 48 h. Note that BAST mhealth was a shorter 10-item version of the original 38-item BAST, a self-report tool to measure the neurobehavioral consequences of TBI. Items for BAST mhealth were selected from the original BAST based on their ability to capture higher more problematic levels of symptoms. For instance, for negative affect, items like “stressed” and “worried” were commonly endorsed by participants, meaning that from an item response theory (IRT) perspective they were likely less useful for capturing higher more problematic (and less common) levels of negative affect [47]. The two items selected to capture negative affect in BAST mhealth asked about the extent to which participants “got mad easily” and “did not enjoy activities that are usually important to me”, as both were considered “high difficulty items” in that only participants with the highest levels of negative affect reported feeling those states. At baseline, information on total number of TBI(s) experienced, total TBIs with loss of consciousness, and worse TBI severity was recorded.

#### 2.2.5. Study 5: First-Year U.S. College Students

The fifth dataset included 525 first-year college students aged 18 to 24 [14]. For two months of their first fall semester, they completed the Perceived Stress Scale (PSS-10) [48] (with reference to the past week) every week and wore the Oura smartring (Oura Health Oy, Oulu, Finland), which records nighttime HRV. Specifically, using machine learning models that take into account heart rate, HRV, and accelerometer data, it records likely periods of sleep, and nighttime HRV was defined as HRV over the longest sleep period recorded for a day [14]. Scores above 14 on the PSS-10 were coded as indicative of moderate-to-high stress [14]. On average participants had 5.9 weeks (SD 1.2) of HRV data recorded.

### 2.3. Statistical Analyses

#### 2.3.1. HRV Metrics

In studies 1 (knowledge workers) and 2 (adults with type 1 diabetes), nighttime HRV was computed based on interbeat intervals (IBIs) recorded on smartwatches between 12 am and 5 am, a timeframe consistent with prior studies [49,50]. The data were first divided into 5 min segments, from which only segments with at least 70% complete IBI data were kept [20]. Next, segments in which any steps were recorded by the smartwatch were removed as the motion could introduce artifacts into the IBI data. The RHRV R package (version 5.0.0) [51] was then used to filter out likely spurious IBI values, interpolate missing values, and compute the following HRV metrics: root mean square of successive differences (RMSSD), standard deviation of normal-to-normal intervals (SDNN), high-frequency (HF) HRV, low-frequency (LF) HRV, and very low frequency (VLF) HRV. RMSSD and SDNN reflect total HRV, though RMSSD is more sensitive to parasympathetic nervous system activity [1]. HRV frequency components capture different regulatory systems influences: HF influences on HR are believed to stem from parasympathetic activity, LF from both parasympathetic and sympathetic activity and baroreceptor activity, and VLF from body temperature regulation and vasomotor activity [1].

In study 3 (student interns), resting HRV was computed based on IBIs recorded over a two-minute period with a heart rate chest strap using the same steps described above. The same HRV metrics as for studies 1 and 2 were computed except for VLF HRV because, for this measure, a minimum heart recording period of five minutes has been recommended [52].

In studies 4 (adults with TBI) and 5 (college students), precomputed resting HRV metrics (i.e., not raw IBI data) were used in analyses as the raw time between heartbeats was not available. Study 4 had RMSSD, SDNN, HF, and LF/HF ratio values for a five-minute period each morning, and study 5 had the weekly average of nighttime RMSSD values (to coincide with their weekly stress measures). The ratio of LF to HF activity has been argued to represent the relative influence of sympathetic activity on heart rate as compared to parasympathetic, but others argue that both LF and HF capture primarily parasympathetic activity making the ratio between them less informative [1].

Among HRV metrics, RMSSD is often preferred due its better statistical properties and strong reflection of total heart rate variability [1]. Therefore, RMSSD was treated as the primary HRV metric in this study. HRV metrics were log transformed when their distributions were not normally distributed (i.e., RMSSD values in studies 3 and 4 were log transformed). Correlations with other HRV metrics were also computed for comprehensiveness.

#### 2.3.2. Correlations Between HRV and Health Variables

Two-level multilevel models were used to compute correlations between HRV and other measures. Multilevel models account for the non-independence in observations resulting from multiple datapoints provided by each individual [53]. They do so by separating within- and between-person effects, or distinguishing between the effect of the long-term average of a measure (e.g., mean stress over the study period) and the effect of deviations from each person’s average levels (e.g., having greater stress than average). Without this distinction, these effects would be conflated. This consideration of different levels results in two types of correlations: within-person correlations here capture associations between daily fluctuations in measures (e.g., more stress in a day would be expected to be associated with lower nighttime HRV), while between-person correlations capture associations between the averages of measures (e.g., greater average stress would be expected to be associated with lower average nighttime HRV).

In the multilevel models used for studies 1 through 4, several days of observations were nested in people. When measures were administered several times daily, their daily average was computed prior to inclusion in correlational analyses. For within-person correlations, consistent with the prior literature [54,55], we expected that poorer scores in any health domain (e.g., greater stress or poorer health behaviors) should be reflected by a lower nighttime HRV or lower next-morning HRV. In terms of between-person correlations, a greater average HRV (recorded at night or in the morning) was expected to be associated with better values in various domains of health (e.g., lower average stress, lower likelihood of diagnoses) [4]. Between-person correlations were adjusted for age and gender because of prior studies suggesting these demographic variables were particularly influential on HRV [52]. Study 5 differed from the others in that each observation did not represent a day but average levels over a week (i.e., average nighttime HRV and stress over a week), but the analyses followed the same multilevel structure. Pearson correlations were computed to examine associations between HRV and continuous variables, while biserial correlations were computed to examine associations between HRV and binary variables. Following convention, correlations of 0.10, 0.30, and 0.50 were considered small, medium, and large effects, respectively [56]. All analyses were conducted using the statistical software M*plus* version 8.11 [57].

#### 2.3.3. Intraclass Correlation Coefficients

Given that within- and between-person associations with HRV are of interest, it may be useful to know the proportion of variance in HRV that is attributable to between-person differences and not daily within-person fluctuations (i.e., intraclass correlation coefficient, or ICC). For instance, if the ICC of HRV is very high, this means that it changes minimally within a person and hence may not be expected to have within-person associations with other measures. To compute ICC, the formula below was used for each HRV metric.

*ICC* = *Var*(*BP*)/(*Var*(*BP*) + *Var*(*WP*))

Var(BP) is the between-person variance in a HRV metric and Var(WP) is the variance of a HRV metric (at the daily level) within a person [58]. ICC values can range from 0 to 1, with 0 indicating that variance of a measure is attributable purely to (daily) within-person fluctuations and 1 indicating that variance is attributable only to between-person differences. For repeated HRV measurement under controlled conditions (i.e., HRV assessment in the laboratory on two separate days), various HRV metrics as measured by ECG has been found to have an ICC of around 0.8 [59,60,61]. To our knowledge, however, the ICC of daily resting HRV measures recorded by wearables in people’s everyday environments have been reported for heart rate chest straps [61] but not other wearables.

## 3. Results

Detailed demographic information is shown in Supplementary Tables S1–S5 for each of the five studies considered in the analyses. The samples were extremely heterogeneous in their sample sizes and participant characteristics. For instance, two of the five studies had clinical samples (i.e., adults with type 1 diabetes and adults with traumatic brain injury). The three non-clinical samples focused on different groups of the general population (i.e., knowledge workers, interns, and college students).

### 3.1. Study 1: U.S. Knowledge Workers (n = 717)

Few of the correlations in study 1 were in the expected directions (see Table 1). At the within-person level, higher positive affect reported on the once daily EMA surveys was unexpectedly associated with significantly lower RMSSD the following night (*r* = −0.02, *p* = 0.034). None of the other within-person correlations with RMSSD were statistically significant. At the between-person level, average RMSSD was not significantly associated with the EMA or baseline measures of mood, trait anxiety, physical activity, or crystallized intelligence. Contrary to expectations, greater mean stress was associated with higher average RMSSD. Consistent with expectations, greater RMSSD was associated with less poor sleep quality (*r* = −0.11, *p* = 0.003) and higher fluid intelligence (*r* = 0.10, *p* = 0.021). RMSSD had an intraclass correlation coefficient (ICC) of 0.68.

### 3.2. Study 2: German Adults with Type 1 Diabetes (n = 108)

The top of Table 2 lists the multilevel correlations between HRV metrics and EMA measures, while the bottom of Table 2 lists correlations between HRV and baseline measures. Within-person EMA correlations were generally in the expected directions but not significant; for instance, greater stress had non-significant associations with lower RMSSD on the following night (*r* = −0.08, *p* = 0.089). At the between-person level, the study average of nighttime HRV metrics was not associated with the study average of any type of stress, mood, or energy.

Several correlations in the expected directions were observed for between-person average nighttime HRV measures and the baseline physiological and self-report measures (bottom of Table 2). Most notably, greater between-person average RMSSD was associated with a lower triglyceride level (*r* = −0.17, *p* = 0.006), lower Hba1c (average blood glucose, *r* = −0.21, *p* = 0.014), greater time in target blood glucose range (*r* = 0.24, *p* = 0.019), fewer depression symptoms (*r* = −0.22, *p* = 0.024), greater resilience (*r* = 0.18, *p* = 0.04), lower diabetes distress (*r* = −0.30, *p* = 0.001), better diabetes self-management (*r* = 0.32, *p* < 0.001), a lower likelihood of neuropathy (*r* = −0.28, *p* = 0.007), and a lower chance of retinopathy (*r* = −0.39, *p* < 0.001). RMSSD had an ICC of 0.70.

### 3.3. Study 3: Student Interns from The Netherlands (n = 25)

In study 3 several within-person correlations between morning HRV metrics (as measured by a chest strap over a two-minute recording period) and EMA surveys aligned with expectations (top of Table 3). At the within-person level, morning RMSSD was not associated with prior-day reports of stress, demands, vigor, or detachment from work; however, still at the within-person level, higher RMSSD was significantly related to more prior-day recovery from work (r = 0.10, *p* = 0.004), less mental exhaustion (r = −0.09, *p* = 0.001), and less alcohol consumption (r = −0.32, *p* = 0.001). The association with self-efficacy on the day prior was in the expected direction but not significant (r = 0.11, *p* = 0.077). Most between-person correlations were non-significant (bottom of Table 3). Contrary to expectation, individuals with a greater average RMSSD over the study reported higher levels of fatigue (r = 0.34, *p* = 0.001), lower happiness (r = −0.37, *p* = 0.014), and lower feelings of fitness (r = −0.29, *p* = 0.042). RMSSD had an ICC of 0.55.

### 3.4. Study 4: U.S. Adults with Lifetime History of Traumatic Brain Injury (n = 55)

In study 4, several correlations between morning HRV metrics (as measured by a chest strap over a five-minute period) and EMA surveys aligned with expectation (Table 4). At the within-person level, higher morning RMSSD was associated with greater executive functioning over the 48 h prior (*r* = 0.10, *p* = 0.037). Associations between morning RMSSD and negative affect (*r* = −0.08, *p* = 0.12) and fatigue (*r* = −0.09, *p* = 0.084) over the past 48 h were in expected directions but non-significant. At the between-person level, greater average morning RMSSD over the study was associated with lower average negative affect (*r* = −0.27, *p* = 0.011), fatigue (*r* = −0.26, *p* = 0.028), fewer TBIs experienced (*r* = −0.30, *p* = 0.003), and fewer TBIs with loss of consciousness (*r* = −0.23, *p* = 0.024). RMSSD had an ICC of 0.62.

### 3.5. Study 5: First-Year U.S. College Students (n = 525)

At the within-person level, average nighttime RMSSD over a week as measured by a smartring was not associated with stress ratings for the corresponding week (Table 5). At the between-person level, average nighttime RMSSD had non-significant relationships with participants’ average raw stress score (*r* = −0.061, *p* = 0.181) and moderate stress (*r* = −0.091, *p* = 0.058) in the expected negative directions. The week average nighttime RMSSD had an ICC of 0.89.

For study specific correlations between health measures and HRV metrics aside from RMSSD, please refer to Supplementary Tables S6–S9. Note that for study 5 it was not possible to compute correlations with HRV metrics aside from RMSSD.

Table 6 summarizes the health domains covered across the 5 HRV studies. Physiological markers were considered in study 2 only, while all studies had at least one variable relevant to physical symptoms and stress at both the within- and between-person levels.

## 4. Discussion

The studies considered in analyses were extremely heterogeneous (e.g., in population and methods of assessment) making identification of patterns difficult, but allowing for examination of correlations between wearable-based resting HRV and a broad array of health domains. Prior research investigated the relationships between wearable-measured HRV and various domains of health in numerous ways [12,13,14,33,41], but re-analyses here provide novel insights specifically on whether measures of different health domains impact *next-day* wearable-measured *resting* HRV within a person, and whether health metrics are associated with average resting HRV across the study. Between-person correlations were small to moderate, while within-person correlations were at most small. Thus, wearable-measured resting HRV appeared more sensitive to trait-like health measures that might differentiate people from each other rather than state-level health measures that might fluctuate within a person. Specifically, the average of several days of resting HRV measures appeared most sensitive to more clinically oriented health measures. Within a person, in one study higher resting HRV was found to be significantly associated with more recovery time from work, less mental exhaustion, and less alcohol consumption on the day prior. Across studies however, within-person correlations with general stress and mood measures from the day prior were non-significant. Findings are summarized in more detail below using the five domains of health covered in the analyzed studies, focusing on correlations with RMSSD.

### 4.1. Mental Health and Emotions

Only in studies 2 and 4 were significant and expected between-person associations observed between resting HRV and mental health. One possible reason for this is that in studies 2 and 4 more clinically oriented mental health questionnaires were used, such as the Patient Health Questionnaire in study 2 and BAST (traumatic brain injury population-focused measure) in study 4. Other studies used measures that captured a broad range of emotional well-being such as PANAS-derived emotion measures [35], not just high negative affect indicative of need for intervention like for clinically oriented tests [42,43]. Perhaps resting HRV is sensitive to high negative affect, but not the broader range of emotional well-being. Consistent with this argument, in study 2, significant between-person associations were found between RMSSD and two clinically oriented baseline measures of depression, but no significant correlations were observed with the study average of a PANAS-like mood item. Contrary to this argument, in study 3, average happiness levels over the study were found to be negatively associated with average resting HRV, but note that this study had a small number of participants (*n* = 25), which is suboptimal for examining between-person relationships. Within-person associations with mental health/emotion measures were generally small (i.e., under *r* = 0.10) and non-significant, and the mental health items were mostly derived from measures without a clinical focus.

### 4.2. Physical Symptoms and Stress

As with the above, HRV appeared to have the most pronounced between-person correlations with more clinically oriented physical symptom and stress measures, and low-to-no between-person associations with less clinically focused measures. Lower average resting HRV was found to be associated with worse sleep quality as measured by the clinically oriented Pittsburgh Sleep Quality Index (study 1, *n* = 717); a greater likelihood of neuropathy and retinopathy (study 2, *n* = 108); greater fatigue as measured by the clinical BAST measure (study 4, *n* = 55); a greater number of past TBIs (study 4); and a greater number of past TBIs with loss of consciousness (study 4) [48]. Correlations ranged in size from *r* = 0.11 (*p* = 0.003) to *r* = 0.39 (*p* < 0.001). Average resting HRV was not found to be associated with the study average of any of the general stress focused measures (studies 1, 2, 3, and 5). Unexpectedly, in study 3, greater average fatigue and lower fitness, as per the less clinically focused measures, were associated with higher mean HRV, but again the small number of participants may have been suboptimal for examining between-person relationships.

At the within-person level, correlations between physical symptoms like stress and resting HRV were typically non-significant and small (i.e., under 0.10) but often in the expected directions. One exception was in study 3, where higher RMSSD was significantly associated with less mental exhaustion on the day prior. Only in study 3 was exhaustion inquired about specifically instead of ‘fatigue’ or ‘energy’. In study 1, which had a large sample size and many observations per individual, momentary stress was asked about only once daily, which may not have been sufficient to represent daily stress. This could have been one factor explaining why for this study the estimated magnitude of the correlation between stress and daily resting HRV was zero.

### 4.3. Health Behaviors

At the between-person level, average resting HRV was not found to be associated with level of physical activity (study 1), smoking status (study 2), or substance misuse (study 4). It was moderately correlated with self-reported diabetes self-management (study 2), a more clinically oriented measure, which is perhaps another example of the trend of greater associations between resting HRV and clinical assessments.

Health behaviors were considered at the within-person level only in studies 3 (*n* = 25, 60 observations per person) and 4 (*n* = 55, 12 observations per person). In study 3, higher morning resting HRV was significantly associated with more recovery time from work (*r* = 0.10, *p* = 0.004) and less alcohol consumption (*r* = −0.32, *p* = 0.001) on the day prior. The within-person correlation with recovery time from work may be small. However, it suggests that resting HRV level could be responsive to work recovery dynamics but is not completely driven by them. In study 4, higher morning resting HRV had a non-significant association with lower substance misuse on the day prior.

### 4.4. Functioning

Higher average nighttime resting HRV was found to have a small significant correlation only with fluid intelligence (study 1). It had small non-significant associations in the expected directions with crystallized intelligence (study 1), self-reported executive functioning (study 4), and impulsivity (study 4).

Only in studies 3 and 4 was functioning measured at the within-person level, and observed correlations were small at most. In study 3, a greater morning resting HRV was not significantly associated with experiencing greater self-efficacy on the day prior, but the effect was in the expected direction. For study 4, a greater morning HRV was associated with greater executive functioning but not impulsivity on the day prior.

### 4.5. Physiological Markers

Physiological measures were assessed only in study 2. At the between-person level, greater average nighttime HRV had near-moderate correlations with higher time in range and lower Hba1c but no associations with any of the other blood glucose metrics. Note that time in range captures glycemic control over the study period, while Hba1c represents average blood glucose over the past 3 months [62]. Thus, HRV appears sensitive to retrospective blood glucose levels (i.e., Haba1c) and immediate glycemic control (i.e., time in range). No significant correlations with cholesterol or inflammatory markers were observed, though estimates were in the expected directions. Greater average nighttime resting HRV was associated with lower triglyceride levels. At the within-person level only blood glucose was assessed, and none of the within-person correlations with blood glucose metrics were found to be significant, perhaps suggesting that a certain level of aggregation of moments of suboptimal glucose levels is required to affect HRV levels.

### 4.6. Intraclass Correlation Coefficients

The ICCs of resting HRV (RMSSD) measures from studies 1 through 4 ranged from 0.55 to 0.70, suggesting that variance in HRV was not solely attributable to between-person influences (i.e., HRV had meaningful fluctuations within a person). These values were lower than the approximately 0.80 ICCs attained from HRV measurement in laboratory conditions [59,60], which is expected given that all HRV measures in this study were recorded for longer periods in people’s natural environments. Study 5 had a higher ICC of 0.89, which likely stemmed from its HRV metrics being computed at the weekly instead of daily level.

### 4.7. Limitations

Various sources of measurement error could have attenuated relationships between HRV and other measures. For instance, for the smartwatch-based nighttime HRV measures used in this study, we operationalized nighttime as between 12 am and 5 am. However, not all participants may have been sleeping during this timeframe. For morning chest strap-based HRV measures, there is uncertainty with regard to how closely people followed the measurement protocol (e.g., participants could have stood up before the measurement duration finished).

The validity of the HRV measures were not examined, which is another important factor that can impact measurement error. Based on prior work, we had made the assumption that both ECG- and PPG-based measures would have greater validity in stationary, as compared to ambulatory, conditions [7,8,9]. However, we did not formally make comparisons to gold-standard HRV measures. Furthermore, a variety of methods could have been used to filter out spurious beats, different criteria for acceptable data could have been implemented (e.g., whether or not stationarity was violated), and different ways of interpolating missing data could have been utilized. These factors could impact the level of validity of the HRV data as well as the percentage of usable data available, but we did not examine them in great detail in this paper.

Possible sample size limitations for studies analyzed here should be noted. In simulations, we found that at least 100 participants are required to detect a between-person correlation of 0.30 with 80% power, and a total of 900 observations (e.g., 100 participants times 9 observations for each) required to detect a within-person correlation of 0.10 with 80% power. Study 2 had 108 participants with an average of 6 nights of HRV data (108 × 6 nights = 648 observations), meaning this study was likely underpowered to detect small statistically significant within-person correlations. Study 3 had 25 participants with an average of 60.0 mornings of HRV recordings (25 participants*60 mornings = 1500 observations), meaning the study was likely underpowered for between-person analyses but sufficiently powered to detect within-person associations. In study 4, 55 participants with approximately 13 observations per person suggested insufficient power to detect small within- or between-person relationships.

Though a large number of correlations was tested, no adjustment for multiple comparisons was made because analyses were more exploratory in nature. It has been argued that when conducting secondary analyses as part of theory building and testing (and without direct treatment implications), the cost of a type II error from a multiple comparisons adjustment can be higher than the cost of type I error as it could lead to failing to pursue potentially fruitful directions of future research [63]. However, to account for the greater possibility of a type I error from not adjusting for multiple comparisons, further less exploratory research is needed to examine if the results can be replicated [64].

An advantage of the diversity of the studies was that a broad array of health domains could be examined, along with different consumer wearables. Consideration of a variety of health domains was an acknowledgement of the multi-faceted nature of health. A disadvantage however was that it was difficult to examine the extent to which findings replicated across studies.

### 4.8. Future Directions

The results of this paper and the study design decisions associated with the datasets used here [12,13,14,33,41] could inform experimental design decisions for future work aiming to more systematically assess the validity of wearable-based resting HRV measures as indicators of general health. For instance, the study results suggest that future investigations on assessing resting HRV with wearables could benefit from being sufficiently powered to detect small associations at both the within- and between-person level (like in studies 1 and 5), including clinically oriented mental health measures (like in studies 2 and 4), including clinically oriented physical health measures (like in all studies aside from study 3), and administering physiological measures (e.g., blood glucose, triglyceride level, cholesterol, and inflammation markers, like in study 2). Ideally, measures of nighttime HRV with a smartwatch, nighttime HRV with a smartring, and morning HRV with a chest strap would all be recorded as there was not enough evidence in this study to dismiss the possibility that any of these measures have relevance to health. If evidence is found supporting all their validities as general markers of general health, then consumers would have more options for methods of measuring HRV in a way that can provide information about health.

We only examined wearable-based resting HRV’s associations with the health domains measured in the included studies. Future studies can examine its associations with other health measures such as blood pressure and variability in symptoms such as pain for a more comprehensive understanding of its health correlates [65].

To help facilitate future research on the health correlates of consumer wearable-measured resting HRV, a first step could be to standardize a terminology for what constitutes resting HRV and health in the wearables context. Resting HRV has often been conceived as the baseline condition in an experiment before exposure to a stimulus [22], but in the context of future work on wearables can adopt the operationalization here of resting HRV as nighttime or waking HRV measures. In clinical medicine health is often understood as the absence of disease [66], but in the context of future work on wearable-measured HRV, health should more broadly encapsulate domains such as mental health, physical health, and physiological health to reflect the diverse health domains that HRV has been associated with in prior work [4,5,6,15] and in this paper.

## 5. Conclusions

Nighttime and morning resting HRV, as assessed by different types of consumer wearables, appeared to have potential to act as indicators of general health (i.e., mental, physical, behavioral, functional, and physiological health) across five heterogeneous studies. They seemed most sensitive to more clinically oriented trait-like (or slow-changing) health measures, but further research is needed to investigate this claim. Future research on consumer wearables that measure HRV may benefit from considering the results of this study for decisions including the type of variables to measure (e.g., triglyceride level, depression, etc.) and the frequency of measurement (e.g., longitudinal assessment to enable analysis of both between- and within-person associations). With continued research in this area, wearable-based resting HRV measurement may be developed to a point where it becomes a widely accessible measure of general health that can inform the health management efforts of individuals and support the health promotion efforts of healthcare providers.

## Figures and Tables

**Table 1:** Multilevel correlations between nighttime HRV metrics and measures of different health domains, with adjustment by age and gender at the between-person level, for study 1 (717 U.S. knowledge workers). Note that all EMA items were administered once daily. Within-person correlations are between health measures and HRV measured the following night.

	RMSSD
**WITHIN-PERSON DAY LEVEL**	
**Mental Health and Emotions**	
Anxiety EMA (“Please select the response that shows how anxious you feel at the moment.”)	0.01 (*p* = 0.29)
Positive Affect EMA (“Respond according to the extent you feel this way in general.”)	−0.02 (*p* = 0.034) *
Negative Affect EMA (Same question as for PA, but different emotion adjectives)	0 (*p* = 0.894)
**Physical Symptoms and Stress**	
Stress EMA (“How would you rate your current level of stress?”)	0 (*p* = 0.767)
**BETWEEN-PERSON**	
**Mental Health and Emotions**	
Anxiety EMA	0.06 (*p* = 0.149)
Positive Affect EMA	−0.06 (*p* = 0.161)
Negative Affect EMA	0.06 (*p* = 0.164)
Pos Affect BSL (Positive and Negative Affect Schedule)	0.02 (*p* = 0.6)
Neg Affect BSL (Positive and Negative Affect Schedule)	0.02 (*p* = 0.597)
Trait Anxiety BSL (State-Trait Anxiety Inventory)	−0.02 (*p* = 0.633)
Neuroticism BSL (Big Five Inventory)	0.03 (*p* = 0.559)
**Physical Symptoms and Stress**	
Stress EMA	0.09 (*p* = 0.031) *
Poor Sleep Quality BSL (Pittsburgh Sleep Quality Index)	−0.11 (*p* = 0.003) *
Significant Sleep Difficulties BSL (1 if has and 0 otherwise)	−0.14 (*p* = 0.001) *
**Health Behaviors**	
Physical Activity BSL (International Physical Activity Questionnaire)	0.02 (*p* = 0.47)
**Functioning**	
Fluid Intelligence BSL (Shipley cognitive functioning)	0.1 (*p* = 0.021) *
Crystallized Intelligence BSL (Shipley cognitive functioning)	0.04 (*p* = 0.315)

Note. BSL: baseline test; EMA: ecological momentary assessment; RMSSD: root mean square of successive differences. * *p* < 0.05.

**Table 2:** The top of Table 2 shows within-person multilevel correlations between nighttime HRV metrics and EMA measures of different health domains, for study 2 (108 German adults with Type 1 diabetes). Note that the stress, mood, and energy EMA items were administered 4 times daily, and all other items were presented once at the end of each day (EOD). Within-person correlations are between daily health measures and HRV measured the following night. The bottom of Table 2 shows between-person correlations between the study-long average of nighttime HRV metrics and measures of different health domains assessed at baseline, with adjustment for age and gender, for study 2 (108 German adults with Type 1 diabetes).

	RMSSD
**WITHIN-PERSON DAY LEVEL**	
**Mental Health and Emotions**	
Mood EMA day average (“How is your mood right now?”)	0.04 (*p* = 0.303)
**Physical Symptoms and Stress**	
Stress EMA day average (“How stressed are you feeling right now?”)	−0.08 (*p* = 0.089)
Energy EMA day average (“How energetic do you feel right now?”)	0.01 (*p* = 0.714)
Work Stress EOD (“Burdened by stress regarding work?”)	−0.06 (*p* = 0.243)
Family Stress EOD (“Burdened by stress regarding close others?)	−0.04 (*p* = 0.383)
Diabetes Stress EOD (“Burdened by stress regarding diabetes?”)	−0.07 (*p* = 0.221)
People Stress EOD	0 (*p* = 0.99)
Diabetes Energy EOD (“Diabetes is taking up too much mental and physical energy?”)	−0.02 (*p* = 0.594)
**Physiological Markers**	
Daily time in range (BG ≥ 70 mg/dL and ≤180 mg/dL)	0.03 (*p* = 0.509)
Daily glucose fluctuations (CV)	0.02 (*p* = 0.665)
Daily hyperglycemia exposure (BG > 180 mg/dL)	−0.03 (*p* = 0.591)
Daily hypoglycemia exposure (BG < 70 mg/dL)	0.03 (*p* = 0.543)
**BETWEEN-PERSON (EMA AND EOD MEASURES)**	
**Mental Health and Emotions**	
Mood EMA	0.04 (*p* = 0.724)
**Physical Symptoms and Stress**	
Stress EMA	0.04 (*p* = 0.692)
Energy EMA	0.06 (*p* = 0.573)
Work Stress EOD	−0.03 (*p* = 0.763)
Family Stress EOD	0.1 (*p* = 0.454)
Diabetes Stress EOD	0.05 (*p* = 0.635)
People Stress EOD	−0.06 (*p* = 0.483)
Diabetes Energy EOD	−0.06 (*p* = 0.546)
**Physiological Markers**	
Daily time in range (BG ≥ 70 mg/dL and ≤180 mg/dL)	0.24 (*p* = 0.019) *
Daily glucose fluctuations (CV)	−0.06 (*p* = 0.569)
Daily hyperglycemia exposure (BG > 180 mg/dL)	−0.07 (*p* = 0.529)
Daily hypoglycemia exposure (BG < 70 mg/dL)	0.04 (*p* = 0.647)
**BETWEEN-PERSON (BASELINE MEASURES)**	
**Mental Health and Emotions**	
Depression (Patient Health Questionnaire)	−0.18 (*p* = 0.049) *
Depression (Center for Epidemiologic Studies Depression Scale)	−0.22 (*p* = 0.024) *
Resilience Scale (RS-13)	0.18 (*p* = 0.04) *
Diabetes distress (Problem Areas in Diabetes Scale)	−0.30 (*p* = 0.001) *
**Physical Symptoms and Stress**	
Neuropathy (1 if has neuropathy and 0 otherwise)	−0.28 (*p* = 0.007) *
Retinopathy (1 if has retinopathy and 0 otherwise)	−0.39 (*p* < 0.001) *
**Health Behaviors**	
Diabetes Self-Management Questionnaire	0.32 (*p* < 0.001) *
Smoker (1 for smoker and 0 otherwise)	−0.12 (*p* = 0.187)
**Physiological Markers**	
Cholesterol	−0.04 (*p* = 0.668)
Triglycerides	−0.17 (*p* = 0.006) *
HDL (High-density lipoprotein cholesterol)	0.13 (*p* = 0.242)
LDL (Low-density lipoprotein cholesterol)	−0.14 (*p* = 0.088)
Hba1c (Hemoglobin A1c)	−0.21 (*p* = 0.014) *
IL6 (Interleukin-6)	−0.10 (*p* = 0.367)
IL10 (Interleukin-10)	−0.04 (*p* = 0.656)
TNF (Tumor necrosis factor)	−0.09 (*p* = 0.059)

Note. BG: blood glucose; CV: coefficient of variation; EMA: ecological momentary assessment; EOD: end of day; RMSSD: root mean square of successive differences. * *p* < 0.05.

**Table 3:** The top of Table 3 shows within-person correlations between morning chest strap-based HRV measures and daily measures of different health domains assessed on the day prior for study 3 (25 student interns from the Netherlands). Happiness, vigor, fatigue, fitness, and self-efficacy were asked about twice daily. All other measures were administered at the end of the day (EOD) or beginning of the day (BOD). The bottom of Table 3 shows between-person correlations between average morning chest strap-based HRV measures over the study and EMA measures of different health domains for study 3, with adjustment for age and gender. Happiness, vigor, fatigue, fitness, and self-efficacy were asked about twice daily. All other measures were administered at the end of the day (EOD) or beginning of the day (BOD).

	Log RMSSD
**WITHIN-PERSON DAY LEVEL**	
**Mental Health and Emotions**	
Happiness EMA day average (“Do you feel happy?”)	−0.04 (*p* = 0.298)
Dedication EOD (“My activities today were full of meaning and purpose”)	−0.02 (*p* = 0.501)
**Physical Symptoms and Stress**	
Demands EOD (“How demanding was your day?”)	−0.03 (*p* = 0.347)
Stress EOD (“How much stress did you perceive today?”)	−0.01 (*p* = 0.755)
Energy EOD (“I felt bursting with energy during my activities.”)	0.05 (*p* = 0.092)
Vigor EMA day average (“Do you feel like undertaking things?”)	−0.01 (*p* = 0.775)
Mental Exhaustion EOD (“I felt mentally exhausted as a result of my activities.”)	−0.09 (*p* = 0.001) *
Subjective sleep BOD (“How was the quality of your sleep?”)	0.13 (*p* = 0.059)
Fatigue EMA day average (“How fatigued do you feel?”)	−0.04 (*p* = 0.407)
Fitness EMA day average (“How fit do you feel?”)	−0.03 (*p* = 0.343)
**Health Behaviors**	
Recovery time EOD (“I had enough time to relax and recover from work.”)	0.10 (*p* = 0.004) *
Detachment EOD (“During my off-job time, I distanced myself from my work.”)	0.03 (*p* = 0.283)
Alcohol consumption day prior BOD (“Yesterday, I consumed “X number” alcoholic beverages.”)	−0.32 (*p* = 0.001) *
**Functioning**	
Self-efficacy EMA day average (“Do you feel capable of solving problems today?”)	0.11 (*p* = 0.077)
**BETWEEN-PERSON**	
**Mental Health and Emotions**	
Happiness EMA day average (“Do you feel happy?”)	−0.37 (*p* = 0.014) *
Dedication EOD (“My activities today were full of meaning and purpose”)	−0.01 (*p* = 0.955)
**Physical Symptoms and Stress**	
Demands EOD (“How demanding was your day?”)	0.02 (*p* = 0.926)
Stress EOD (“How much stress did you perceive today?”)	−0.01 (*p* = 0.972)
Energy EOD (“I felt bursting with energy during my activities.”)	−0.16 (*p* = 0.456)
Vigor EMA day average (“Do you feel like undertaking things?”)	−0.30 (*p* = 0.179)
Mental Exhaustion EOD (“I felt mentally exhausted as a result of my activities.”)	−0.16 (*p* = 0.368)
Subjective sleep BOD (“How was the quality of your sleep?”)	0.13 (*p* = 0.436)
Fatigue EMA day average (“How fatigued do you feel?”)	0.41 (*p* < 0.001) *
Fitness EMA day average (“How fit do you feel?”)	−0.29 (*p* = 0.042) *
**Health Behaviors**	
Recovery time EOD (“I had enough time to relax and recover from work.”)	−0.10 (*p* = 0.569)
Detachment EOD (“During my off-job time, I distanced myself from my work.”)	−0.10 (*p* = 0.501)
Alcohol consumption day prior BOD (“Yesterday, I consumed “X number” alcoholic beverages.”)	−0.04 (*p* = 0.811)
**Functioning**	
Self-efficacy EMA day average (“Do you feel capable of solving problems today?”)	−0.11 (*p* = 0.505)

Note. BOD: beginning of day; EMA: ecological momentary assessment; EOD: end of day; RMSSD: root mean square of successive differences. * *p* < 0.05.

**Table 4:** Multilevel correlations between morning chest strap-based HRV metrics and measures of different health domains, with adjustment for age and gender, for study 4 (55 U.S. adults with past traumatic brain injury). Within-person correlations are between morning HRV and same-day health measures covering the last 48 h.

	Log RMSSD
**WITHIN-PERSON DAY LEVEL**	
**Mental Health and Emotions**	
Negative affect EMA (“I got mad easily” and “I did not enjoy activities that are usually important to me”)	−0.08 (*p* = 0.12)
**Physical Symptoms and Stress**	
Fatigue EMA (“I felt too tired to finish tasks that required thinking” and “I had low energy”)	−0.09 (*p* = 0.084)
**Health Behaviors**	
Substance misuse EMA	−0.04 (*p* = 0.148)
**Functioning**	
Executive function EMA (“I started activities on my own” and “I was organized”)	0.10 (*p* = 0.037) *
Impulsivity EMA (“I acted rudely” and “I took unnecessary risks”)	−0.01 (*p* = 0.806)
**BETWEEN-PERSON**	
**Mental Health and Emotions**	
Negative affect EMA	−0.27 (*p* = 0.011) *
**Physical Symptoms and Stress**	
Fatigue EMA	−0.26 (*p* = 0.028) *
Total TBI(s) experienced BSL	−0.30 (*p* = 0.003) *
Total TBI(s) with LOC BSL	−0.23 (*p* = 0.024) *
Worst injury severity ^a^ BSL	0.02 (*p* = 0.899)
**Health Behaviors**	
Substance misuse EMA	0.11 (*p* = 0.4)
**Functioning**	
Executive function EMA	0.22 (*p* = 0.068)
Impulsivity EMA	−0.06 (*p* = 0.659)

Note. BSL: baseline; EMA: ecological momentary assessment; LOC: loss of consciousness; RMSSD: root mean square of successive differences; TBI: traumatic brain injury. ^a^ 5-point ordinal scale, with a score of 1 indicating no TBI history, scores of 2 to 3 considered mild TBI, and scores of 4 to 5 considered moderate-severe TBI. * *p* < 0.05.

**Table 5:** Multilevel correlations between nighttime smartring-based HRV metrics and stress, with adjustment for gender, for study 5 (525 first-year U.S. college students). Age was not adjusted for because it was not provided, but all participants were roughly the same age (i.e., 18 to 24 years). Within-person correlations are between average nighttime HRV over a week and stress items asked with reference to the same week.

	RMSSD
**WITHIN-PERSON**	
**Physical Symptoms and Stress**	
Perceived Stress Scale (referring to the past week)	−0.01 (*p* = 0.606)
Moderate stress (binary) ^a^	−0.04 (*p* = 0.070)
**BETWEEN-PERSON**	
**Physical Symptoms and Stress**	
Perceived Stress Scale	−0.061 (*p* = 0.181)
Moderate stress (binary)	−0.091 (*p* = 0.058)

Note. RMSSD: root mean square of successive differences. ^a^ Scores above 14 on the PSS were considered indicative of moderate-to-high stress.

**Table 6:** Summary of health domains covered across the 5 wearable-measured HRV studies.

	Mental Health and Emotions		Physical Symptoms and Stress		Health Behaviors		Functioning		Physiological Markers	
	With	Btw	With	Btw	With	Btw	With	Btw	With	Btw
Study 1: U.S. Knowledge Workers (*n* = 717)	X	X	X	X		X		X		
Study 2: German Adults with Type 1 Diabetes (*n* = 108)	X	X	X	X		X			X	X
Study 3: Student Interns from the Netherlands (*n* = 25)	X	X	X	X	X	X	X	X		
Study 4: U.S. Adults with Lifetime History of Traumatic Brain Injury (*n* = 55)	X	X	X	X	X	X	X	X		
Study 5: First-Year U.S. College Students (*n* = 525)			X	X						

Note. With: within-person relationships between HRV and health analyzed; Btw: between-person relationships analyzed.

## Data Availability

Data available on request due to restrictions (e.g., privacy, legal or ethical reasons). Code used to compute nighttime resting HRV from the smartwatch recorded interbeat intervals and code for statistical analyses are available at https://osf.io/nj3cq/ (accessed on 16 November 2025).

## Supplementary Materials

The following supporting information can be downloaded at: https://www.mdpi.com/article/10.3390/s25237147/s1.
