# Supplementary material for: Resting Heart Rate Variability Measured by Consumer Wearables and Its Associations with Diverse Health Domains in Five Longitudinal Studies
Source: Sensors (Basel). 2025 Nov 22;25(23):7147. doi: 10.3390/s25237147 (PMC12693838; doi:10.3390/s25237147)
Supplement: Supplementary file 1 [file sensors-25-07147-s001.zip › sensors-3924280-supplementary.pdf]

Supplementary Material

Supplementary Table S1. Demographic information for study 1, which focused on U.S. knowledge workers (n=717).

Characteristic	Mean (SD) or Percentage (%)
Age range (years)	
<25	87 (12.1)
25-34	319 (44.5)
35-44	190 (26.5)
45-54	88 (12.3)
55-64	32 (4.5)
Sex	
Female	293 (40.9)
Male	424 (59.1)
Working Status	
Employed	717 (100)
Not employed	0 (0)

Supplementary Table S2. Demographic information for study 2, which focused on German Adults with Type 1 Diabetes (n=108).

**Characteristic**	**Mean (SD) or Percentage (%)**
Age (years)	39.7 (12.6)
Sex	
Male	42 (38.9)
Female	66 (61.1)
Employment	
Worker	83 (76.9)
Non-worker	25 (23.1)
Country of residence	
Germany	98 (90.7)
Other	10 (9.3)
Smoking	
Smoker	30 (27.8)
Non-smoker	78 (72.2)
Neuropathy	
Past or current neuropathy	38 (35.2)
No neuropathy	70 (64.8)
Retinopathy	
Past or current retinopathy	25 (23.1)
No retinopathy	83 (76.9)

Supplementary Table S3. Demographic information for study 3, which focused on student interns from the Netherlands (n=25).

Characteristic	Mean (SD) or Percentage (%)
Age (years)	23.3 (2.9)
Sex	
Male	2 (8)
Female	23 (92)

Supplementary Table S4. Demographic information for study 4, which focused on U.S. adults with past traumatic brain injury (n=55).

Characteristic	Mean (SD) or Percentage (%)
Age (years)	43.2 (14.2)
Sex	
Male	17 (30.9)
Female	38 (69.1)
Education	
High school or lower	3 (5.5)
Some College	11 (20)
Bachelor's degree	13 (23.6)
Graduate degree	28 (50.9)
TBI History	
No TBI	11 (20)
Prior TBI	44 (80)

Supplementary Table S5. Demographic information for study 5, which focused on first year U.S. college students (n=525).

Characteristic	Mean (SD) or Percentage (%)
Sex	
Male	144 (27.4)
Female	368 (70.1)
Transgender and Nonbinary	13 (2.5)
Race	
Non-White	65 (12.4)
White	460 (87.6)

Supplementary Table S6. Multilevel correlations between nighttime HRV metrics and measures of different health domains, with adjustment by age and gender at the between level, for study 1 (717 U.S. knowledge workers). Note that all EMA items were administered once daily. Within person correlations are between health measures and HRV measured the following night.

	RMSSD	SDNN	Log HF	Log LF	Log VLF
**WITHIN-PERSON DAY LEVEL**					
**Mental Health and Emotions**					
Anxiety EMA ("Please select the response that shows how anxious you feel at the moment.")	0.01 (*P*=.29)	0.01 (*P*=.049)*	0.01 (*P*=.369)	0.01 (*P*=.402)	0.01 (*P*=.177)
Positive Affect EMA ("Respond according to the extent you feel this way in general.")	-0.02 (*P*=.034)*	0 (*P*=.822)	-0.03 (*P*<.001)*	-0.03 (*P*<.001)*	-0.04 (*P*<.001)*
Negative Affect EMA (Same question as for PA, but different emotion adjectives)	0 (*P*=.894)	0 (*P*=.829)	0.00 (*P*=.848)	0.00 (*P*=.602)	0.00 (*P*=.758)
**Physical Symptoms and Stress**					
Stress EMA ("How would you rate your current level of stress?")	0 (*P*=.767)	0 (*P*=.681)	0.00 (*P*=.655)	0.00 (*P*=.775)	0.00 (*P*=.619)
**BETWEEN-PERSON**					
**Mental Health and Emotions**					
Anxiety EMA	0.06 (*P*=.149)	0.08 (*P*=.051)	0.03 (*P*=.486)	0.09 (*P*=.038)*	0.10 (*P*=.019)*
Positive Affect EMA	-0.06 (*P*=.161)	-0.07 (*P*=.064)	-0.04 (*P*=.395)	-0.04 (*P*=.419)	-0.03 (*P*=.467)
Negative Affect EMA	0.06 (*P*=.164)	0.05 (*P*=.216)	0.06 (*P*=.084)	0.05 (*P*=.237)	0.06 (*P*=.144)
Pos Affect BSL (Positive and Negative Affect Schedule)	0.02 (*P*=.6)	0.03 (*P*=.438)	0.00 (*P*=.914)	0.01 (*P*=.884)	0.00 (*P*=.964)
Neg Affect BSL (Positive and Negative Affect Schedule)	0.02 (*P*=.597)	0.04 (*P*=.345)	0.03 (*P*=.407)	0.02 (*P*=.666)	0.02 (*P*=.636)
Trait Anxiety BSL (State-Trait Anxiety Inventory)	-0.02 (*P*=.633)	0 (*P*=.93)	-0.01 (*P*=.708)	-0.02 (*P*=.687)	0.00 (*P*=.936)
Neuroticism BSL (Big Five Inventory)	0.03 (*P*=.559)	0.04 (*P*=.367)	0.04 (*P*=.325)	0.01 (*P*=.874)	0.02 (*P*=.622)
**Physical Symptoms and Stress**					
Stress EMA	0.09 (*P*=.031)*	0.09 (*P*=.027)*	0.06 (*P*=.144)	0.12 (*P*=.003)*	0.12 (*P*=.005)*
Poor Sleep Quality BSL (Pittsburgh Sleep Quality Index)	-0.11 (*P*=.003)*	-0.09 (*P*=.029)*	-0.12 (*P*=.004)*	-0.12 (*P*=.004)*	-0.14 (*P*=.001)*

Significant Sleep Difficulties BSL (1 if has and 0 otherwise)	-0.14 (*P*=.001)*	-0.12 (*P*=.004)*	-0.15 (*P*<.001)*	-0.16 (*P*<.001)*	-0.17 (*P*<.001)*
**Health Behaviours**					
Physical Activity BSL (International Physical Activity Questionnaire)	0.02 (*P*=.47)	0.05 (*P*=.234)	0.01 (*P*=.708)	0.04 (*P*=.29)	0.02 (*P*=.669)
**Functioning**					
Fluid Intelligence BSL (Shipley cognitive functioning)	0.1 (*P*=.021)*	0.07 (*P*=.089)	0.08 (*P*=.054)	0.06 (*P*=.165)	0.04 (*P*=.36)
Crystallized Intelligence BSL (Shipley cognitive functioning)	0.04 (*P*=.315)	0.04 (*P*=.307)	0.01 (*P*=.709)	0.07 (*P*=.092)	0.04 (*P*=.276)

Note. BSL:baseline test; EMA:ecological momentary assessment; HF: High-frequency HRV; LF: Low-frequency HRV; RMSSD: Root Mean Square of Successive Differences; SDNN: Standard Deviation of Normal-to-Normal intervals; VLF: Very low-frequency HRV.

*p<0.05

RMSSD, SDNN, HF, LF, and VLF had intraclass correlation coefficients (ICC) of 0.68, 0.61, 0.66, 0.73, and 0.66 respectively.

Supplementary Table S7. The top of Table 2 shows within-person multilevel correlations between nighttime HRV metrics and EMA measures of different health domains for study 2 (108 German adults with Type 1 diabetes). Note that the stress, mood, and energy EMA items were administered 4 times daily, and all other items were presented once at the end of each day (EOD). Within person correlations are between daily health measures and HRV measured the following night. The bottom of Table 2 shows between-person correlations between study long average of nighttime HRV metrics and measures of different health domains assessed at baseline, *with adjustment for age and gender*, for study 2 (108 German adults with Type 1 diabetes).

	RMSSD	SDNN	Log HF	Log LF	Log VLF
**WITHIN-PERSON DAY LEVEL**					
**Mental Health and Emotions**					
Mood EMA day average (“How is your mood right now?”)	0.04 (*P*=.303)	0.01 (*P*=.902)	0.06 (*P*=.207)	0.02 (*P*=.776)	0.06 (*P*=.292)
**Physical Symptoms and Stress**					
Stress EMA day average (“How stressed are you feeling right now?”)	-0.08 (*P*=.089)	-0.02 (*P*=.587)	-0.09 (*P*=.065)	-0.04 (*P*=.445)	-0.07 (*P*=.168)
Energy EMA day average (“How energetic do you feel right now?”)	0.01 (*P*=.714)	0.04 (*P*=.359)	0.02 (*P*=.704)	-0.01 (*P*=.775)	0.02 (*P*=.722)
Work Stress EOD (“Burdened by stress regarding work?”)	-0.06 (*P*=.243)	-0.1 (*P*=.03)*	-0.09 (*P*=.091)	-0.10 (*P*=.076)	-0.11 (*P*=.062)
Family Stress EOD (“Burdened by stress regarding close others?)	-0.04 (*P*=.383)	0 (*P*=.992)	-0.05 (*P*=.233)	-0.06 (*P*=.186)	-0.04 (*P*=.464)
Diabetes Stress EOD (“Burdened by stress regarding diabetes?”)	-0.07 (*P*=.221)	0 (*P*=.977)	-0.03 (*P*=.58)	-0.01 (*P*=.884)	-0.01 (*P*=.843)
People Stress EOD	0 (*P*=.99)	-0.03 (*P*=.378)	-0.04 (*P*=.369)	-0.01 (*P*=.785)	-0.05 (*P*=.385)
Diabetes Energy EOD (“Diabetes is taking up too much mental and physical energy?”)	-0.02 (*P*=.594)	-0.03 (*P*=.49)	0.00 (*P*=.992)	0.05 (*P*=.244)	-0.02 (*P*=.659)
**Physiological Markers**					
Daily time in range (BG>=70 mg/dL and <=180 mg/dL)	0.03 (*P*=.509)	-0.06 (*P*=.242)	0.03 (*P*=.278)	0.03 (*P*=.414)	0.04 (*P*=.286)
Daily glucose fluctuations (CV)	0.02 (*P*=.665)	0 (*P*=.945)	0.00 (*P*=.939)	-0.02 (*P*=.715)	-0.02 (*P*=.647)
Daily hyperglycaemia exposure (BG >180 mg/dL)	-0.03 (*P*=.591)	0.05 (*P*=.238)	-0.04 (*P*=.378)	-0.01 (*P*=.79)	0.01 (*P*=.63)
Daily hypoglycaemia exposure (BG <70 mg/dL)	0.03 (*P*=.543)	0.02 (*P*=.627)	0.00 (*P*=.955)	-0.01 (*P*=.735)	-0.03 (*P*=.445)
**BETWEEN-PERSON (EMA AND EOD MEASURES)**					
**Mental Health and Emotions**					

Mood EMA	0.04 (*P*=.724)	0.03 (*P*=.783)	-0.03 (*P*=.808)	0.07 (*P*=.524)	0.06 (*P*=.523)
**Physical Symptoms and Stress**					
Stress EMA	0.04 (*P*=.692)	0.05 (*P*=.625)	0.06 (*P*=.596)	0.02 (*P*=.866)	0.01 (*P*=.894)
Energy EMA	0.06 (*P*=.573)	0.05 (*P*=.607)	0.01 (*P*=.894)	0.08 (*P*=.47)	0.06 (*P*=.546)
Work Stress EOD	-0.03 (*P*=.763)	0.02 (*P*=.806)	-0.01 (*P*=.942)	-0.05 (*P*=.585)	-0.03 (*P*=.735)
Family Stress EOD	0.1 (*P*=.454)	0.14 (*P*=.236)	0.13 (*P*=.262)	0.06 (*P*=.558)	0.05 (*P*=.604)
Diabetes Stress EOD	0.05 (*P*=.635)	0.02 (*P*=.823)	0.12 (*P*=.293)	0.07 (*P*=.525)	0.05 (*P*=.593)
People Stress EOD	-0.06 (*P*=.483)	-0.03 (*P*=.777)	-0.04 (*P*=.679)	-0.05 (*P*=.595)	-0.01 (*P*=.937)
Diabetes Energy EOD	-0.06 (*P*=.546)	-0.06 (*P*=.544)	-0.01 (*P*=.923)	-0.06 (*P*=.598)	-0.06 (*P*=.546)
**Physiological Markers**					
Daily time in range (BG>=70 mg/dL and <=180 mg/dL)	0.24 (*P*=.019)*	0.23 (*P*=.026)*	0.30 (*P*=.002)*	0.22 (*P*=.016)*	0.29 (*P*=.001)*
Daily glucose fluctuations (CV)	-0.06 (*P*=.569)	-0.04 (*P*=.691)	-0.04 (*P*=.701)	-0.01 (*P*=.931)	0.00 (*P*=.959)
Daily hyperglycemia exposure (BG >180 mg/dL)	-0.07 (*P*=.529)	0.02 (*P*=.842)	-0.09 (*P*=.432)	-0.04 (*P*=.694)	-0.08 (*P*=.461)
Daily hypoglycemia exposure (BG <70 mg/dL)	0.04 (*P*=.647)	0.06 (*P*=.456)	0.11 (*P*=.269)	0.10 (*P*=.263)	0.14 (*P*=.098)
**BETWEEN-PERSON (BASELINE MEASURES)**					
**Mental Health and Emotions**					
Depression (Patient Health Questionnaire)	-0.18 (*P*=.049)*	-0.18 (*P*=.048)*	-0.04 (*P*=.614)	-0.10 (*P*=.244)	-0.10 (*P*=.19)
Depression (Center for Epidemiologic Studies Depression Scale)	-0.22 (*P*=.024)*	-0.23 (*P*=.018)*	-0.12 (*P*=.235)	-0.11 (*P*=.264)	-0.14 (*P*=.162)
Resilience Scale (RS-13)	0.18 (*P*=.04)*	0.09 (*P*=.355)	0.04 (*P*=.685)	0.04 (*P*=.68)	0.08 (*P*=.393)
Diabetes distress (Problem Areas in Diabetes Scale)	-0.30 (*P*=.001)*	-0.21 (*P*=.038)*	-0.18 (*P*=.074)	-0.16 (*P*=.147)	-0.17 (*P*=.089)
**Physical Symptoms and Stress**					
Neuropathy (1 if has neuropathy and 0 otherwise)	-0.28 (*P*=.007)*	-0.26 (*P*=.016)*	-0.27 (*P*=.004)*	-0.29 (*P*=.003)*	-0.27 (*P*=.007)*
Retinopathy (1 if has retinopathy and 0 otherwise)	-0.39 (*P*<.001)*	-0.38 (*P*<.001)*	-0.42 (*P*<.001)*	-0.49 (*P*<.001)*	-0.48 (*P*<.001)*
**Health Behaviours**					
Diabetes Self-Management Questionnaire	0.32 (*P*<.001)*	0.20 (*P*=.03)*	0.28 (*P*=.002)*	0.19 (*P*=.032)*	0.18 (*P*=.056)

Smoker (1 for smoker and 0 otherwise)	-0.12 (*P*=.187)	-0.06 (*P*=.595)	-0.15 (*P*=.143)	-0.15 (*P*=.144)	-0.17 (*P*=.108)
**Physiological Markers**					
Cholesterol	-0.04 (*P*=.668)	-0.04 (*P*=.694)	-0.04 (*P*=.722)	-0.03 (*P*=.781)	-0.05 (*P*=.62)
Triglycerides	-0.17 (*P*=.006)*	-0.22 (*P*=.013)*	-0.23 (*P*=.016)*	-0.23 (*P*=.018)*	-0.21 (*P*=.047)*
HDL (High-density lipoprotein cholesterol)	0.13 (*P*=.242)	0.17 (*P*=.073)	0.17 (*P*=.08)	0.17 (*P*=.031)*	0.14 (*P*=.064)
LDL (Low-density lipoprotein cholesterol)	-0.14 (*P*=.088)	-0.13 (*P*=.069)	-0.16 (*P*=.109)	-0.21 (*P*=.073)	-0.19 (*P*=.085)
Hba1c (Hemoglobin A1c)	-0.21 (*P*=.014)*	-0.18 (*P*=.087)	-0.28 (*P*=.005)*	-0.2 (*P*=.044)*	-0.25 (*P*=.009)*
IL6 (Interleukin-6)	-0.10 (*P*=.367)	-0.04 (*P*=.769)	-0.08 (*P*=.447)	-0.04 (*P*=.644)	-0.02 (*P*=.83)
IL10 (Interleukin-10)	-0.04 (*P*=.656)	-0.04 (*P*=.723)	-0.06 (*P*=.547)	-0.05 (*P*=.51)	-0.10 (*P*=.161)
TNF (Tumor necrosis factor)	-0.09 (*P*=.059)	-0.02 (*P*=.78)	-0.02 (*P*=.806)	-0.07 (*P*=.282)	-0.09 (*P*=.093)

Note. BG: blood glucose; CV: coefficient of variation; EMA: ecological momentary assessment; EOD: end of day; HF: High-frequency HRV; LF: Low-frequency HRV; RMSSD: Root Mean Square of Successive Differences; SDNN: Standard Deviation of Normal-to-Normal intervals; VLF: Very low-frequency HRV.

*p<0.05

RMSSD, SDNN, HF, LF, and VLF had ICCs of 0.70, 0.68, 0.71, 0.81, and 0.82 respectively.

Supplementary Table S8. The top of Table 3 shows within-person correlations between morning chest strap based HRV measures and daily measures of different health domains assessed on the day prior for study 3 (25 student interns from the Netherlands). Happiness, vigour, fatigue, fitness, and self-efficacy were asked about twice daily. All other measures were administered at the end of the day (EOD) or beginning of the day (BOD). The bottom of Table 3 shows between-person correlations between average morning chest strap based HRV measures over the study and EMA measures of different health domains for study 3 (25 student interns from the Netherlands), with adjustment for age and gender. Happiness, vigour, fatigue, fitness, and self-efficacy were asked about twice daily. All other measures were administered at the end of the day (EOD) or beginning of the day (BOD).

	**Log RMSSD**	**Log SDNN**	**Log HF**	**Log LF**
**WITHIN-PERSON DAY LEVEL**				
**Mental Health and Emotions**				
Happiness EMA day average (“Do you feel happy?”)	-0.04 (*P*=.298)	-0.06 (*P*=.171)	-0.04 (*P*=.373)	0.00 (*P*=.935)
Dedication EOD (“My activities today were full of meaning and purpose”)	-0.02 (*P*=.501)	-0.04 (*P*=.113)	-0.03 (*P*=.319)	-0.06 (*P*=.049)*
**Physical Symptoms and Stress**				
Demands EOD (“How demanding was your day?”)	-0.03 (*P*=.347)	-0.05 (*P*=.191)	-0.03 (*P*=.447)	-0.06 (*P*=.2)
Stress EOD (“How much stress did you perceive today?”)	-0.01 (*P*=.755)	-0.02 (*P*=.491)	0.00 (*P*=.979)	-0.07 (*P*=.031)*
Energy EOD (“I felt bursting with energy during my activities.”)	0.05 (*P*=.092)	0.05 (*P*=.026)*	0.02 (*P*=.5)	0.03 (*P*=.236)
Vigor EMA day average (“Do you feel like undertaking things?”)	-0.01 (*P*=.775)	-0.01 (*P*=.765)	0.00 (*P*=.926)	-0.01 (*P*=.877)
Mental Exhaustion EOD (“I felt mentally exhausted as a result of my activities.”)	-0.09 (*P*=.001)*	-0.08 (*P*=.002)*	-0.08 (*P*=.002)*	-0.08 (*P*=.018)*
Subjective sleep BOD (“How was the quality of your sleep?”)	0.13 (*P*=.059)	0.13 (*P*=.036)*	0.11 (*P*=.087)	0.09 (*P*=.045)*
Fatigue EMA day average (“How fatigued do you feel?”)	-0.04 (*P*=.407)	-0.04 (*P*=.313)	-0.03 (*P*=.54)	-0.07 (*P*=.011)*
Fitness EMA day average (“How fit do you feel?”)	-0.03 (*P*=.343)	-0.05 (*P*=.026)*	-0.04 (*P*=.316)	-0.04 (*P*=.147)
**Health Behaviours**				
Recovery time EOD (“I had enough time to relax and recover from work.”)	0.10 (*P*=.004)*	0.08 (*P*=.039)*	0.10 (*P*<.001)*	0.10 (*P*=.004)*
Detachment EOD (“During my off-job time, I distanced myself from my work.”)	0.03 (*P*=.283)	0.01 (*P*=.715)	0.04 (*P*=.129)	0.04 (*P*=.324)
Alcohol consumption day prior BOD (“Yesterday, I consumed “X number” alcoholic beverages.”)	-0.32 (*P*=.001)*	-0.28 (*P*<.001)*	-0.28 (*P*=.003)*	-0.20 (*P*=.001)*
**Functioning**				
Self-efficacy EMA day average (“Do you feel capable of solving problems today?”)	0.11 (*P*=.077)	0.06 (*P*=.298)	0.11 (*P*=.075)	0.10 (*P*=.023)*
**BETWEEN-PERSON**				
**Mental Health and Emotions**				
Happiness EMA day average (“Do you feel happy?”)	-0.37 (*P*=.014)*	-0.31 (*P*=.032)*	-0.43 (*P*=.003)*	-0.21 (*P*=.174)

Dedication EOD ("My activities today were full of meaning and purpose")	-0.01 (*P*=.955)	-0.07 (*P*=.754)	-0.03 (*P*=.884)	-0.06 (*P*=.787)
**Physical Symptoms and Stress**				
Demands EOD ("How demanding was your day?")	0.02 (*P*=.926)	-0.08 (*P*=.676)	0.03 (*P*=.904)	-0.11 (*P*=.568)
Stress EOD ("How much stress did you perceive today?")	-0.01 (*P*=.972)	-0.13 (*P*=.502)	0.06 (*P*=.819)	-0.12 (*P*=.424)
Energy EOD ("I felt bursting with energy during my activities.")	-0.16 (*P*=.456)	-0.08 (*P*=.702)	-0.19 (*P*=.356)	-0.08 (*P*=.697)
Vigor EMA day average ("Do you feel like undertaking things?")	-0.30 (*P*=.179)	-0.27 (*P*=.231)	-0.42 (*P*=.034)*	-0.15 (*P*=.48)
Mental Exhaustion EOD ("I felt mentally exhausted as a result of my activities.")	-0.16 (*P*=.368)	-0.30 (*P*=.041)*	-0.12 (*P*=.577)	-0.37 (*P*=.005)*
Subjective sleep BOD ("How was the quality of your sleep?")	0.13 (*P*=.436)	0.18 (*P*=.25)	0.10 (*P*=.564)	0.22 (*P*=.184)
Fatigue EMA day average ("How fatigued do you feel?")	0.41 (*P*<.001)*	0.30 (*P*=.011)*	0.50 (*P*<.001)*	0.19 (*P*=.136)
Fitness EMA day average ("How fit do you feel?")	-0.29 (*P*=.042)*	-0.20 (*P*=.197)	-0.37 (*P*=.016)*	-0.08 (*P*=.641)
**Health Behaviours**				
Recovery time EOD ("I had enough time to relax and recover from work.")	-0.10 (*P*=.569)	-0.08 (*P*=.614)	-0.12 (*P*=.523)	0.03 (*P*=.842)
Detachment EOD ("During my off-job time, I distanced myself from my work.")	-0.10 (*P*=.501)	-0.06 (*P*=.7)	-0.16 (*P*=.342)	0.08 (*P*=.62)
Alcohol consumption day prior BOD ("Yesterday, I consumed "X number" alcoholic beverages.")	-0.04 (*P*=.811)	-0.08 (*P*=.595)	0.01 (*P*=.955)	-0.16 (*P*=.276)
**Functioning**				
Self-efficacy EMA day average ("Do you feel capable of solving problems today?")	-0.11 (*P*=.505)	-0.09 (*P*=.62)	-0.21 (*P*=.188)	-0.04 (*P*=.814)

Note. BOD: beginning of day; EMA: ecological momentary assessment; EOD: end of day; RMSSD: Root Mean Square of Successive Differences; SDNN: Standard Deviation of Normal-to-Normal intervals.
*p<0.05
RMSSD, SDNN, HF, and LF had ICCs of 0.55, 0.56, 0.44, and 0.51 respectively.

Supplementary Table S9. Multilevel correlations between morning chest strap based HRV metrics and measures of different health domains, with adjustment for age and gender, for study 4 (55 U.S. adults with past traumatic brain injury). Within person correlations are between morning HRV and same day health measures covering the last 48 hours.

	**Log RMSSD**	**Log SDNN**	**Log HF**	**Log LFHF**
**WITHIN-PERSON DAY LEVEL**				
**Mental Health and Emotions**				
Negative affect EMA ("I got mad easily" and "I did not enjoy activities that are usually important to me")	-0.08 (*P*=.12)	-0.10 (*P*=.096)	-0.05 (*P*=.318)	0.00 (*P*=.943)
**Physical Symptoms and Stress**				
Fatigue EMA ("I felt too tired to finish tasks that required thinking" and "I had low energy")	-0.09 (*P*=.084)	-0.06 (*P*=.343)	-0.11 (*P*=.029)*	0.10 (*P*=.021)*
**Health Behaviours**				
Substance misuse EMA	-0.04 (*P*=.148)	-0.11 (*P*=.002)*	-0.01 (*P*=.585)	-0.03 (*P*=.188)
**Functioning**				
Executive function EMA ("I started activities on my own" and "I was organized")	0.10 (*P*=.037)*	0.05 (*P*=.211)	0.11 (*P*=.008)*	-0.05 (*P*=.173)
Impulsivity EMA ("I acted rudely" and "I took unnecessary risks")	-0.01 (*P*=.806)	-0.03 (*P*=.427)	0.02 (*P*=.718)	-0.01 (*P*=.748)
**BETWEEN-PERSON**				
**Mental Health and Emotions**				
Negative affect EMA	-0.27 (*P*=.011)*	-0.16 (*P*=.082)	-0.23 (*P*=.065)	0.28 (*P*=.014)*
**Physical Symptoms and Stress**				
Fatigue EMA	-0.26 (*P*=.028)*	-0.19 (*P*=.05)	-0.29 (*P*=.023)*	0.17 (*P*=.263)
Total TBI(s) experienced BSL	-0.30 (*P*=.003)*	-0.15 (*P*=.095)	-0.26 (*P*=.003)*	0.25 (*P*=.016)*
Total TBI(s) with LOC BSL	-0.23 (*P*=.024)*	-0.05 (*P*=.674)	-0.19 (*P*=.038)*	0.14 (*P*=.211)
Worst injury severity[a] BSL	0.02 (*P*=.899)	0.11 (*P*=.33)	0.00 (*P*=.977)	0.17 (*P*=.22)
**Health Behaviours**				
Substance misuse EMA	0.11 (*P*=.4)	0.12 (*P*=.205)	0.06 (*P*=.638)	-0.03 (*P*=.786)
**Functioning**				
Executive function EMA	0.22 (*P*=.068)	0.12 (*P*=.281)	0.20 (*P*=.099)	-0.15 (*P*=.353)
Impulsivity EMA	-0.06 (*P*=.659)	0.08 (*P*=.515)	-0.08 (*P*=.566)	0.25 (*P*=.041)*

Note. BSL: baseline; EMA: ecological momentary assessment; HF: High-frequency HRV; LFHF: Low-frequency to high-frequency HRV ratio; LOC: loss of consciousness; RMSSD: Root Mean Square of Successive Differences; SDNN: Standard Deviation of Normal-to-Normal intervals; TBI: traumatic brain injury.

[a]5-point ordinal scale, with a score of 1 indicating no TBI history, scores of 2 to 3 considered mild TBI, and scores of 4 to 5 considered moderate-severe TBI; *p<0.05

RMSSD, SDNN, HF, and LFHF had ICCs of 0.62, 0.65, 0.60, and 0.50 respectively.
